# Supplementary material for: “There is a strangeness in this disease”: A qualitative study of parents’ experiences caring for a child diagnosed with COVID-19
Source: PLoS One. 2024 Apr 2;19(4):e0300146. doi: 10.1371/journal.pone.0300146 (PMC10986929; doi:10.1371/journal.pone.0300146)
Supplement: S1 File — (DOCX) [file pone.0300146.s001.docx]

**Supporting Information File 1**

**Screening Criteria**

May 2020- January 2021, parents were asked the following via email:

- We are currently looking to interview parents whose children have been diagnosed with COVID. Do you meet that description?

In January 2021, parents were asked the following via email:

- We are currently looking to in interview Canadian parents whose children have been diagnosed with COVID-19. Do you meet that description?

In September 2021, parents were asked the following via email:

- We are currently looking to interview Canadian parents whose children have been diagnosed with COVID since Fall 2021. Do you meet that description?

In February 2022, parents were asked the following via email:

- We are currently looking to interview Canadian parents whose child was hospitalized with COVID-19. Do you meet that description?
