# Supplementary material for: “There is a strangeness in this disease”: A qualitative study of parents’ experiences caring for a child diagnosed with COVID-19
Source: PLoS One. 2024 Apr 2;19(4):e0300146. doi: 10.1371/journal.pone.0300146 (PMC10986929; doi:10.1371/journal.pone.0300146)
Supplement: S2 File — (DOCX) [file pone.0300146.s002.docx]

**Supporting Information File 2**

**Demographics Questions**

**Please Tell Us About Yourself. Please place a check-mark on your response.**

1) a. Which is your gender?

□ Male

□ Female

□ Other:

2) What is your relationship to the child diagnosed with COVID-19?

□ Parent

□ Grandparent

□ Other family member

□ Guardian

3) What is your Age?

□ Less than 20 years old

□ 20-30 years

□ 31-40 years

□ 41-50 years

□ 51 years and older

4) What is your Marital Status?

□ Married/Partnered

□ Single

5) What is your gross annual household income?

□ Less than $25,000

□ $25,000-$49,999

□ $50,000-$74,999

□ $75,000-$99,999

□ $100,000-$149,999

□ $150,000 and over

□ Prefer not to answer

6) What is your highest level of education?

□ Some high school

□ High school diploma

□ Some post-secondary

□ Post-secondary certificate/diploma

□ Post-secondary degree

□ Graduate degree

□ Other

7) How many children do you have? ­­­­_______

10) What is the age of the child diagnosed with COVID-19? ______

11) a. Does the child diagnosed with COVID-19 have any other diagnosed health conditions?

□ Yes

□ No

b. If yes, what are they? ________________

12) a. Did the child diagnosed with COVID-19 require care from an Emergency Department?

□ Yes

□ No

13) b. Was the child diagnosed with COVID-19 admitted to a hospital as a result of the infection?

□ Yes

□ No
