# Supplementary material for: “There is a strangeness in this disease”: A qualitative study of parents’ experiences caring for a child diagnosed with COVID-19
Source: PLoS One. 2024 Apr 2;19(4):e0300146. doi: 10.1371/journal.pone.0300146 (PMC10986929; doi:10.1371/journal.pone.0300146)
Supplement: S3 File — (DOCX) [file pone.0300146.s003.docx]

**Supporting Information File 3**

**Interview Guide**

**Understanding the Information Needs & Experiences of Parents with a child with COVID19**

Parents will be interviewed to understand their information needs and experience having a child diagnosed with COVID19. Semi-structured interviews will be conducted with parents in order to get their “narrative” or experiences. The following questions will be used to guide these interviews. Being true to semi-structured interview techniques, interview questions will start broad and then move to the more specific. Also, after every interview, the interviewer will reflect on the interview guide to see if any new information emerged during the interview that would suggest that the interview guide needs to be adapted moving forward.

1. Tell me about your experience having your child diagnosed with COVID19
   1. Probe for:
      1. the events leading up to your child being diagnosed. (Do they know how/when they became infected)
      2. signs/symptoms your child was experiencing (Child’s health)
         1. Child’s age
      3. visits to healthcare professionals or telehealth to understand what was happening. (Probe number of visits/telehealth, types of healthcare settings they visited, what did they do? Medications, assessments; were other diagnoses given to your child?)
      4. things you tried at home to decrease the symptoms (did you ask family members, friends, public health, grandparents for help to understand what could be wrong with the child).
      5. Did you look for information at this time? (where did you look for information)
      6. how did your child feel during this time? (emotions, mental state)
      7. how did you feel during this time? (emotions, mental state)
2. Tell me when your child was diagnosed with COVID.
   1. Probe for:
      1. who diagnosed your child with COVID?
      2. did your child go to the Emergency Department? Where were they diagnosed
      3. how did they diagnosis your child with COVID19 (what tests did they do? How long did you have to wait to receive the test results? How did you feel while you were waiting?)
3. Tell me what happened after they were diagnosed with COVID19?
   1. Probe for:
      1. What medical care did you your child receive (hospitalized? Went to ED, went to family physician, etc)?
      2. Sense of child’s trajectory of illness (resp status, energy level, difficulty breathing, appetite, other symptoms)
      3. medications ordered? (tell me about the medications and response to medications)
      4. education given for day-to-day management
      5. changes that the family needed to make for the child with COVID19.
4. Tell me about the when your child was able to go home.
   1. When did your child get discharged home? How did you feel about managing your child’s health needs – Probe for parent’s level of comfort/knowledge/skill being able to manage their child’s needs.
5. Tell me about the rest of your family and how they were affected with your child’s diagnosis of COVID19.
   1. Probe for:
      1. Did the family need to make significant lifestyle or living arrangement changes? (quarantine/self isolation; how did you and your family manage this; how was it having parents and children in the home together for so long).
      2. Did other family members get COVID19? How did you protect others from getting sick?
      3. How did the diagnosis affect parent/caregiver work?
      4. How did other family members (especially other children) react to the diagnosis? What challenges were associated with this?
6. How did your child adapt to having COVID?
   1. Probe for:
      1. Isolation at home/hospital
      2. Mental/psychology impact of having COVID19
      3. Not seeing friends/family members
      4. No school/sports/other outside interests
7. Do you feel that you had adequate knowledge about COVID19 to understand what was happening to your child and to manage their illness?
8. Where did you look for COVID19 information?
   1. Probe for what their preferred sources and formats of health information are.
9. Were all of your questions/info needs about COVID19 answered?
10. Was there information that you wished you had about COVID19 but could not find? Tell me about that.
