# Supplementary material for: “There is a strangeness in this disease”: A qualitative study of parents’ experiences caring for a child diagnosed with COVID-19
Source: PLoS One. 2024 Apr 2;19(4):e0300146. doi: 10.1371/journal.pone.0300146 (PMC10986929; doi:10.1371/journal.pone.0300146)
Supplement: Attachment — Submitted filename: Response to Reviewers.docx [file pone.0300146.s004.docx]

Dear Editor Miwako Hosoda:

Thank you for your insightful comments and suggestions. Please see below for our responses to each of the following comments.

| **Editor comments** | **Author response** |
| --- | --- |
| 1. Please ensure that your manuscript meets PLOS ONE's style requirements, including those for file naming. | We have revised our manuscript to meet PLOS ONE’s style requirements. Thank you. |
| 2. We note that you have indicated that data from this study are available upon request. PLOS only allows data to be available upon request if there are legal or ethical restrictions on sharing data publicly. For more information on unacceptable data access restrictions, please see <http://journals.plos.org/plosone/s/data-availability#loc-unacceptable-data-access-restrictions>.  In your revised cover letter, please address the following prompts:  a) If there are ethical or legal restrictions on sharing a de-identified data set, please explain them in detail (e.g., data contain potentially sensitive information, data are owned by a third-party organization, etc.) and who has imposed them (e.g., an ethics committee). Please also provide contact information for a data access committee, ethics committee, or other institutional body to which data requests may be sent.  b) If there are no restrictions, please upload the minimal anonymized data set necessary to replicate your study findings as either Supporting Information files or to a stable, public repository and provide us with the relevant URLs, DOIs, or accession numbers. For a list of acceptable repositories, please see <http://journals.plos.org/plosone/s/data-availability#loc-recommended-repositories>.  We will update your Data Availability statement on your behalf to reflect the information you provide. | We have revised our cover letter to include details on the ethical restrictions for data being available upon request. Please see the second paragraph of our revised cover letter. Thank you. |
| 3. Please review your reference list to ensure that it is complete and correct. If you have cited papers that have been retracted, please include the rationale for doing so in the manuscript text, or remove these references and replace them with relevant current references. Any changes to the reference list should be mentioned in the rebuttal letter that accompanies your revised manuscript. If you need to cite a retracted article, indicate the article’s retracted status in the References list and also include a citation and full reference for the retraction notice. | We have updated our reference list in track changes in our revised manuscript submission to reflect the updated references added to our manuscript at this stage of revisions. No papers have been retracted. |
| I think this is a very significant paper that interviews and carefully analyzes the various difficulties that parents of children affected by COVID-19 feel.  However, it would be required to show some robust theoretical frameworks such as Uncertainty and Health Literacy, with references provided. | Thank you for this comment and for providing valuable feedback on the use of uncertainty and health literacy theoretical frameworks. We did not set an a-priori theoretical framework to interpret or analyze our findings. While we did not utilize uncertainty and health literacy theory to underpin our philosophical and methodological position from the start, we do believe these frameworks provide useful context to ground our findings. Therefore, we have now expanded our discussion section to include these theories, please see track changes on pages 19, 20, 21. Particularly, Brashers (2001) conceptualization of uncertainty was referenced to introduce uncertainty and provide further context on uncertainty in relation to heath literacy and information (see page 19). Hogg’s (2007) uncertainty-identity theory, originating from psychology, was cited to illuminate the links between the uncertain conditions produced by COVID-19 and parents’ experiences of wanting a sense of community (see page 19-20). On pages 20 and 21, other works were cited to provide uncertainty within the context of COVID-19 and our findings. |
| The survey included those who have children under the age of 18. Were there differences in conclusions based on the age of the children? Parents' opinions may vary depending on whether they are babies, toddlers, children, or teenagers. | Thank you for this comment. While this is indeed an important question to ask, the purpose of our research question was to explore the experiences and information needs of parents of children diagnosed with COVID-19 throughout the pandemic. Our aim was not to conduct a comparative analysis of children by age or development age, and therefore, understanding differences based on age is not within the purpose or scope of this study. |
