# Supplementary material for: “There is a strangeness in this disease”: A qualitative study of parents’ experiences caring for a child diagnosed with COVID-19
Source: PLoS One. 2024 Apr 2;19(4):e0300146. doi: 10.1371/journal.pone.0300146 (PMC10986929; doi:10.1371/journal.pone.0300146)
Supplement: Attachment — Submitted filename: Response to Reviewers (1).docx [file pone.0300146.s005.docx]

Dear Editor:

Thank you for your insightful comments and suggestions. Please see below for our responses to each of the following comments.

| **Journal and Editor comments** | **Author response** |
| --- | --- |
| 1. Please review your reference list to ensure that it is complete and correct. If you have cited papers that have been retracted, please include the rationale for doing so in the manuscript text, or remove these references and replace them with relevant current references. Any changes to the reference list should be mentioned in the rebuttal letter that accompanies your revised manuscript. If you need to cite a retracted article, indicate the article’s retracted status in the References list and also include a citation and full reference for the retraction notice. | No additional references were added to our reference list. No papers have been retracted. |
| 2. If you have data on the age of the children, please could you include this in the findings and discussion as the age of the child could have a massive effect on the experience of the parents. An example could be a baby that has developed a severe cough could be more alarming than in a 17 year old. This would not lead to a comparison of findings, it would just provide extra context that influences the parents' experiences and could influence the future development of a knowledge translation tool. | Thank you for this comment. We have added in data on the age group of the children in the findings; see parent quotations throughout findings on pages 10 – 18 (e.g., interview 4, parents of teenager). We have revised our methods section to reflect these changes (i.e., included the number of children in the family); see page 6 lines 98 to 100. |
