# Supplementary material for: “There is a strangeness in this disease”: A qualitative study of parents’ experiences caring for a child diagnosed with COVID-19
Source: PLoS One. 2024 Apr 2;19(4):e0300146. doi: 10.1371/journal.pone.0300146 (PMC10986929; doi:10.1371/journal.pone.0300146)
Supplement: Attachment — Submitted filename: Response to Reviewers_August 2023-2.docx [file pone.0300146.s006.docx]

Dear Editor,

Thank you for your insightful comments and suggestions. Please see below for our responses to each of the reviewer/editor comments.

| Reviewer Comments | Response and revisions made |
| --- | --- |
| Thank you for including the age of the child in the results section. However, please include a discussion of the influence of the age of the child in the discussion section. Did you notice a difference in for example the findings around anxiety based on the age of the child? Did the age of the child appear to influence any of the other themes and if it did, how does this compare with previous literature? Would you have any different recommendations based on this? | Thank you for your comments about the age of the children with COVID-19. In response to this comment we have:  -added Table 2 (lines 146) which delineates the ages of the children with COVID-19  -Lines 493 – we have added that “parents reported anxiety regardless of the age of their child with COVID-19”  -Lines 503 – we have added “There was little difference in parents’ anxiety based on the age of their child however parents’ reported that their anxiety was tied to different events (e.g., online learning).  -Line 565 – In the limitation section we added that comparing parental experiences based upon the ages of the child with COVID-19 extended beyond the primary focus of this paper. |
