# Supplementary material for: “There is a strangeness in this disease”: A qualitative study of parents’ experiences caring for a child diagnosed with COVID-19
Source: PLoS One. 2024 Apr 2;19(4):e0300146. doi: 10.1371/journal.pone.0300146 (PMC10986929; doi:10.1371/journal.pone.0300146)
Supplement: Attachment — Submitted filename: Response to Reviewers_29JAN2024.docx [file pone.0300146.s007.docx]

Dear Editor:

Thank you for your insightful comments and suggestions. Please see below for our responses to each of the following comments.

| **Journal and Editor comments** | **Author response** |
| --- | --- |
| 1. Please provide a checklist such as SRQR or COREQ | Please find attached the completed COREQ checklist. Based on the COREQ we added additional text to the manuscript to address the following items:  Item 2 we have added our credentials  Item 3 – we have added our occupations  Item 7 – we added in reasons for doing the research  Item 8 - we added in our interests in the research topic  Item 20 – we added in our use of field notes  Item 22 – data saturation was addressed |
| 2. Missing is a discussion about trustworthiness of data and transferability of findings | We have addressed trustworthiness by clarifying the terminology in use of the four pillars as per Lincoln and Guba (1986). This is on line 127 page 7. We have added in a statement addressing transferability on line 132, page 7  In addition we have added in 3 additional references to our methodology: 1 to strengthen the sample methodology section (page 5 line 88)  And 2 to strengthen data collection section (page 6 line 98 and line 115) |
