# Supplementary material for: “There is a strangeness in this disease”: A qualitative study of parents’ experiences caring for a child diagnosed with COVID-19
Source: PLoS One. 2024 Apr 2;19(4):e0300146. doi: 10.1371/journal.pone.0300146 (PMC10986929; doi:10.1371/journal.pone.0300146)
Supplement: Attachment — Submitted filename: Response to Reviewers_20FEB2024.docx [file pone.0300146.s008.docx]

Dear Editor:

Thank you for your insightful comments and suggestions. Please see below for our responses to each of the following comments.

| **Journal and Editor comments** | **Author response** |
| --- | --- |
| 1. Please add heading study design to the methods section in accordance with COREQ | We have added this to page 5 |
| 2. Illustrate an example of your analysis from code, subtheme to theme in a table | Please find attached Table 2: analysis example |
| 3. Please add a results table that illustrate themes and their respective subthemes | Please find attached Table 3: results table |
